# MYH9 is crucial for stem cell-like properties in non-small cell lung cancer by activating mTOR signaling

**DOI:** 10.1038/s41420-021-00681-z

**Published:** 2021-10-11

**Authors:** Meng Chen, Li-Xin Sun, Long Yu, Jun Liu, Li-Chao Sun, Zhi-Hua Yang, Xiong Shu, Yu-Liang Ran

**Affiliations:** 1 State Key Laboratory of Molecular Oncology, National Cancer Center/National Clinical Research Center for Cancer/Cancer Hospital, Chinese Academy of Medical Sciences and Peking Union Medical College, Beijing, China; 2 Beijing Research Institute of Orthopaedics and Traumatology, Beijing JiShuiTan Hospital, Beijing, China

**Keywords:** Cancer stem cells, Molecular biology

## Abstract

The fatality rate of non-small cell lung cancer (NSCLC) has been high due to the existence of cancer stem cells (CSCs). Non-muscle myosin heavy chain 9 (MYH9) can promote the progression of various tumors, but its effect on the stem cell-like characteristics of lung cancer cells (LCCs) has not been clarified. Our research found that the stemness characteristics of LCCs were significantly enhanced by the overexpression of MYH9, and the knockout of MYH9 had the opposite effects. The in vivo with inhibitor blebbistatin further confirmed the effect of MYH9 on the stem cell-like behavior of LCCs. Furthermore, western blotting showed that the expression level of CSCs markers (CD44, SOX2, Nanog, CD133, and OCT4) was also regulated by MYH9. Mechanistic studies have shown that MYH9 regulates stem cell-like features of LCCs by regulating the mTOR signaling pathway, which was supported by sphere formation experiments after LCCs were treated with inhibitors Rapamycin and CHIR-99021. Importantly, high expression of MYH9 in lung cancer is positively correlated with poor clinical prognosis and is an independent risk factor for patients with NSCLC.

## Introduction

Lung cancer has always been considered a serious disease characterized by poor prognosis and a low survival rate. The morbidity and mortality of lung cancer remain high, which is closely related to the prevalence of tobacco and the deterioration of the environment [1, 2]. Despite the continuous progress in diagnosis and treatment, the number of deaths from lung cancer is still increasing, mainly caused by the recurrence, metastasis, and drug resistance of lung cancer.

The occurrence and maintenance of lung cancer depend on the presence of lung cancer stem cells (LCSCs), which contribute to the poor prognosis of patients with lung cancer [3–6]. In 2005, Kim et al. found that the bronchiolar alveolar stem cells isolated from the lung injury model were the source cells of lung adenocarcinoma [7]. In 2007, Ho et al. isolated side population cells with strong tumorigenicity, self-renewal ability, invasion ability, and drug resistance from LCCs, and proved that these cells are the initial cells of lung cancer [8]. Subsequently, researchers found CD133, CD44, and other LCSC surface markers and related signal pathways [9–13], further confirming the existence of LCSCs. At present, various targeted drugs for cancer stem cells (CSCs) have entered phase I or phase II clinical trials [14–16], which is expected to bring a breakthrough in cancer therapy. However, because of the complexity of the structure and diversity of lung tumor tissue subtypes, it is difficult to identify specific targets for LCSCs. Thus, the new therapeutic targets for LCSCs have become an urgent goal in lung cancer treatment.

As a skeleton-related protein, non-muscle myosin heavy chain 9 (MYH9) is widely expressed in cells and tissues and belongs to a kind of nonmuscle myosin II (non-muscle myosin, NM II) [17]. MYH9 can hydrolyze ATP, convert chemical energy into mechanical motion and participate in various processes that require contractile force, such as cell division, migration, shape maintenance, and signal transduction [18, 19]. MYH9 mutations lead to autosomal-dominant diseases, kidney diseases, and thrombotic diseases [20–23] and participate in the occurrence and development of a variety of tumors. The researchers found that the highly expressed MYH9 was involved in the metastasis and recurrence of gastric, colorectal, renal, esophageal, and breast cancer [24–28]. The high expression of MYH9 in NSCLC was found by individual researchers [29]. Some data also show that MYH9 can affect the maintenance characteristics of mesenchymal stem cells and gastrointestinal epithelial stem cells [30, 31]. However, research on LCSCs is lacking, researchers know little about its function in the process of NSCLC, the specific molecular and related signal pathway regulation mechanisms have not been clarified.

In the research, we analyzed the stemness regulatory function of MYH9 on LCCs and its mechanism, and found that MYH9 enhanced the stem cell-like biological behavior of LCCs by activating the mTOR signaling pathway. Also, we used an immunohistochemical (IHC) assay to analyze the expression of MYH9 in clinical tissue samples of lung cancer and found that it can be used as an independent factor in evaluating the prognosis of patients with NSCLC.

## Results

### Enrichment and expression of MYH9 in spheroid cells of lung cancer

**Fig. 1:**
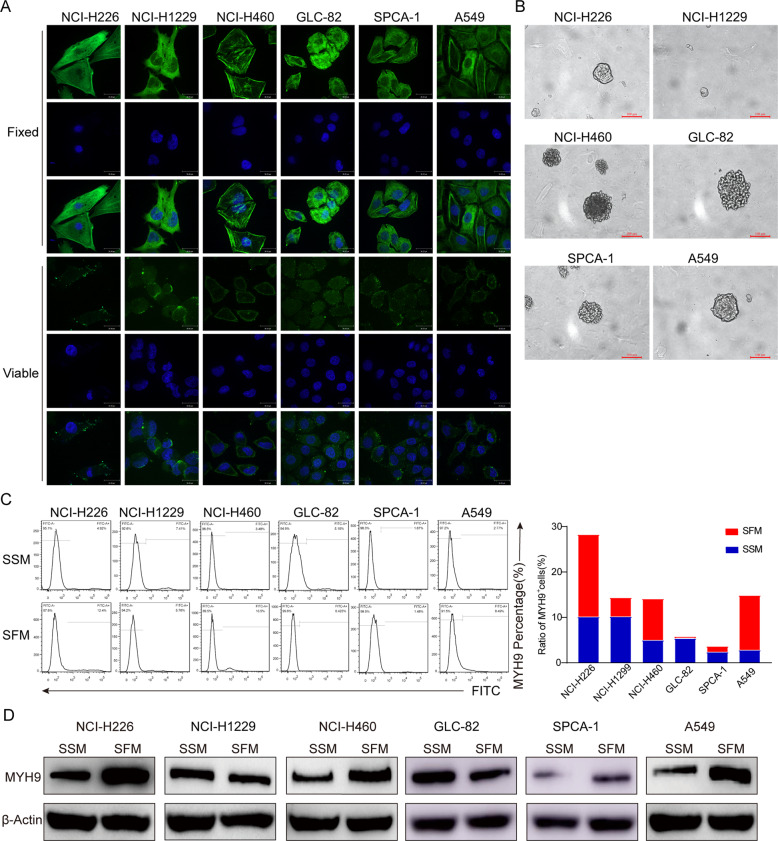
Enrichment and expression of MYH9 in spheroid cells of lung cancer. **A** Expression of MYH9 in lung cancer cell lines (GLC-82, SPCA-1, NCI-H460, A549, NCI-H226, and NCI-H1299) (scale bar, 30 μm). **B** Serum-free suspension culture of LCCs. **C** Analysis of the proportion of MYH9-positive cells in parent and spheroid cells of lung cancer by flow fluorescence. **D** The expression level of MYH9 in parent and spheroid cells of lung cancer cell lines. Data are expressed as mean ± SEM of three independent experiments. *p < 0.05, **p < 0.01, compared to SSM.

To study the expression and localization of MYH9 in NSCLC cell lines, we performed a live-cell and fixed-cell immunofluorescence assay used in six cell lines, namely NCI-H460, SPCA-1, A549, NCI-H1299, NCI-H226, and GLC-82. MYH9 was expressed in the cytoplasm, membrane, and nucleus of different LCCs (Fig. 1A). Serum-free enrichment culture in vitro is a simple and reliable method for screening CSCs. The results showed that the spheroid formed by A549, NCI-H460, and NCI-H226 exhibited a tightened appearance (Fig. 1B). Subsequently, the expression of MYH9 from parent and spheroid cells in different LCCS was studied by flow fluorescence assay. Results (Fig. 1C) showed that, the proportion of MYH9 in spheroid cells from NCI-H226, NCI-H460, and A549 were 12.4%, 10.5%, and 8.49%, respectively, whereas in the parent cells were 4.92%, 3.48%, and 2.77%, respectively. The positive expression rate of spheroid cells was 2.5, 3.0, and 3.1 times that of parent cells, respectively. The expression of MYH9 in spheroid cells from NCI-H226, NCI-H460, and A549 was upregulated (Fig. 1D) compared with parent cells. These results suggest that MYH9 is expressed in the membrane, cytoplasm, and nucleus of LCCs and is enriched in spheroid cells, which may identify LCSCs.

### Downregulation of MYH9 expression suppresses the stem cell-like malignant phenotype of LCCs in vivo and in vitro

**Fig. 2:**
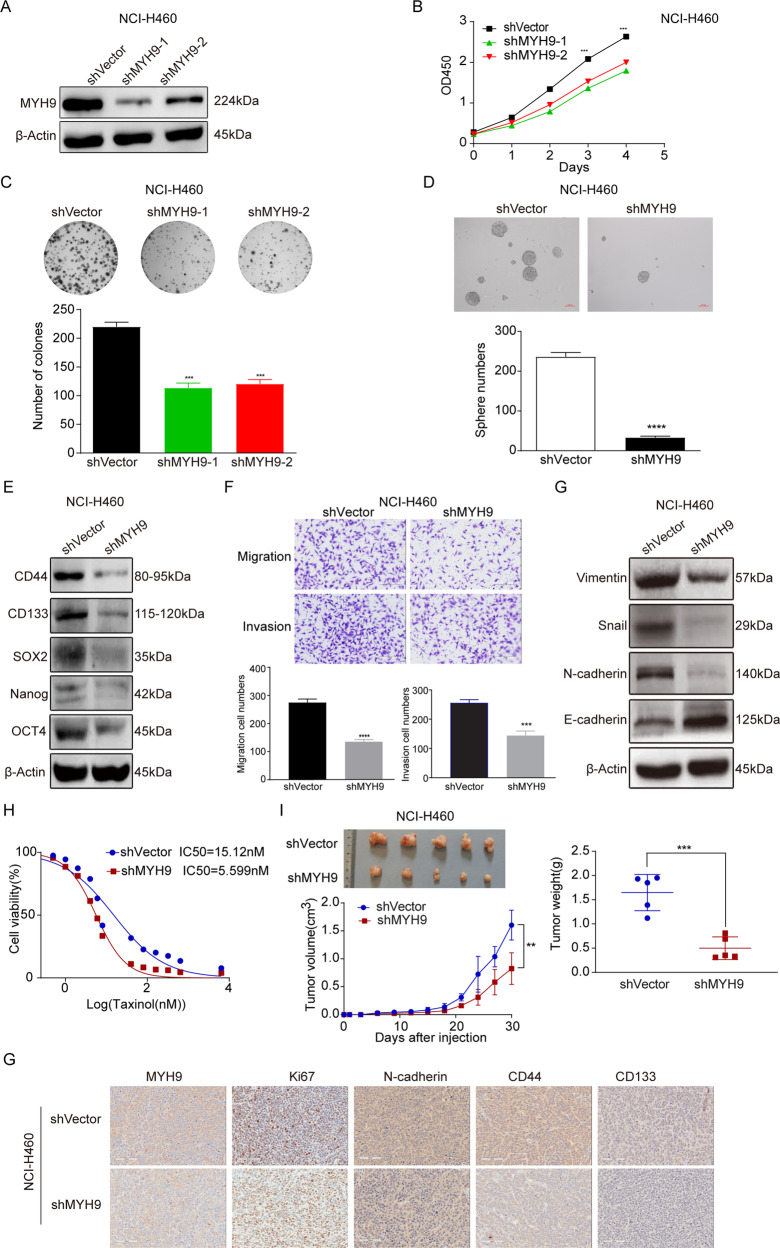
Downregulation of MYH9 expression suppresses the stem cell-like malignant phenotype of LCCs in vivo and in vitro. **A** The protein expression of MYH9 in NCI-H460-shMYH9 stable transformant and control cells. **B**, **C** Proliferation ability of NCI-H460-shMYH9 and NCI-H460-shVector cells (mean ± SD; *n* = 3; ***p < 0.001; Student’s *t*-test). **D** Test results of self-renewal ability of NCI-H460-shMYH9 and NCI-H460-shVector cells (mean ± SD; *n* = 3; ****p < 0.0001; Student’s *t*-test). **E** The expression of CD44, SOX2, CD133, OCT4, and Nanog in NCI-H460-shMYH9 and NCI-H460-shVector cells. **F** Analysis of migration and invasion ability of MYH9 stable knockdown and control cells (scale bar, 200 μm; mean ± SD; n = 3; ***p < 0.001 and ****p < 0.0001; Student’s *t*-test). **G** Western blot of EMT marker in NCI–H460–shMYH9 and NCI-H460-shVector cells. **H** Analysis of drug resistance of NCI-H460-shMYH9 and NCI-H460-shVector cells to paclitaxel. **I** Analysis of the effect of MYH9 knockout on the growth of LCCs in vivo by subcutaneous tumor formation assay (mean ± SD; *n* = 5; **p < 0.01 and ***p < 0.001; Student’s *t*-test). **J** IHC analysis of N-cadherin, MYH9, CD133, CD44, and Ki67 in xenografts of NCI-H460-shMYH9 and NCI-H460-shVector cells (scale bar, 100 μm; *n* = 5).

To study the effect of MYH9 on biological behavior related to the stemness of LCCs, we selected NCI-H460 with high endogenous expression of MYH9 to construct the knockdown stable transformation cell line (Fig. 2A). The clone formation assay is used to detect the regulatory role of MYH9 on the proliferation of LCCs. The results showed that the proliferation ability of MYH9 knockdown cells decreased significantly in vitro (Fig. 2B, C). To explore the sphere-forming ability of MYH9 knockdown cells, we found that the expression of MYH9 was down-regulated, the number of sphere-forming cells decreased significantly, and the inhibition rate of sphere-forming ability reached 86% in NCI-H460-shMYH9 cells (Fig. 2D). Compared with the control cells, the protein expression levels of CD44, CD133, SOX2, Nanog, and OCT4 in MYH9 knockdown cells were decreased (Fig. 2E), indicating that MYH9 affected the expression of stemness-related genes.

The ability of invasion and migration plays an important role in the metastasis and recurrence of tumor. The migration and invasion ability of MYH9 knockdown cells was significantly weaker than that of control cells (Fig. 2F). Western blot also detected the expression of EMT marker in MYH9 knockdown cells. After the downregulation of MYH9, E-cadherin was significantly upregulated, whereas the expression of N-cadherin, Vimentin, and Snail was significantly downregulated (Fig. 2G), indicating that MYH9 promotes the invasion and migration of LCCs. Drug resistance is an important malignant phenotype of CSCs. The IC50 values of MYH9 knockdown cells and control cells to paclitaxel were 5.599 nM and 15.12 nM (Fig. 2H), respectively, indicating that the sensitivity of LCCs to paclitaxel increased with the decrease of MYH9.

Tumorigenicity is one of the most important characteristics of CSCs. To observe the effect of MYH9 on the growth of LCCs in vivo, cells stably knocked down by MYH9 grew slowly in nude mice, and the volume and weight of xenografts were significantly smaller than those in the control group (Fig. 2I). Also, IHC was performed to verify the role of MYH9 in the growth, invasion, and stemness of lung cancer xenografts. The expression of MYH9 in knockout xenografts was lower than that in the control group, and the expression of Ki67 was also significantly downregulated.

In contrast, the results of N-cadherin, CD44, and CD133 were consistent with Western blot. After the knockout of MYH9, the expression in xenografts decreased (Fig. 2J). These results showed that the malignant phenotype of tumors decreased after the down-regulated expression of MYH9, indicating that MYH9 affects the development of lung cancer by regulating LCSC characteristics.

### Overexpression of MYH9 enhances the stemness characteristics of LCCs in vivo and in vitro

**Fig. 3:**
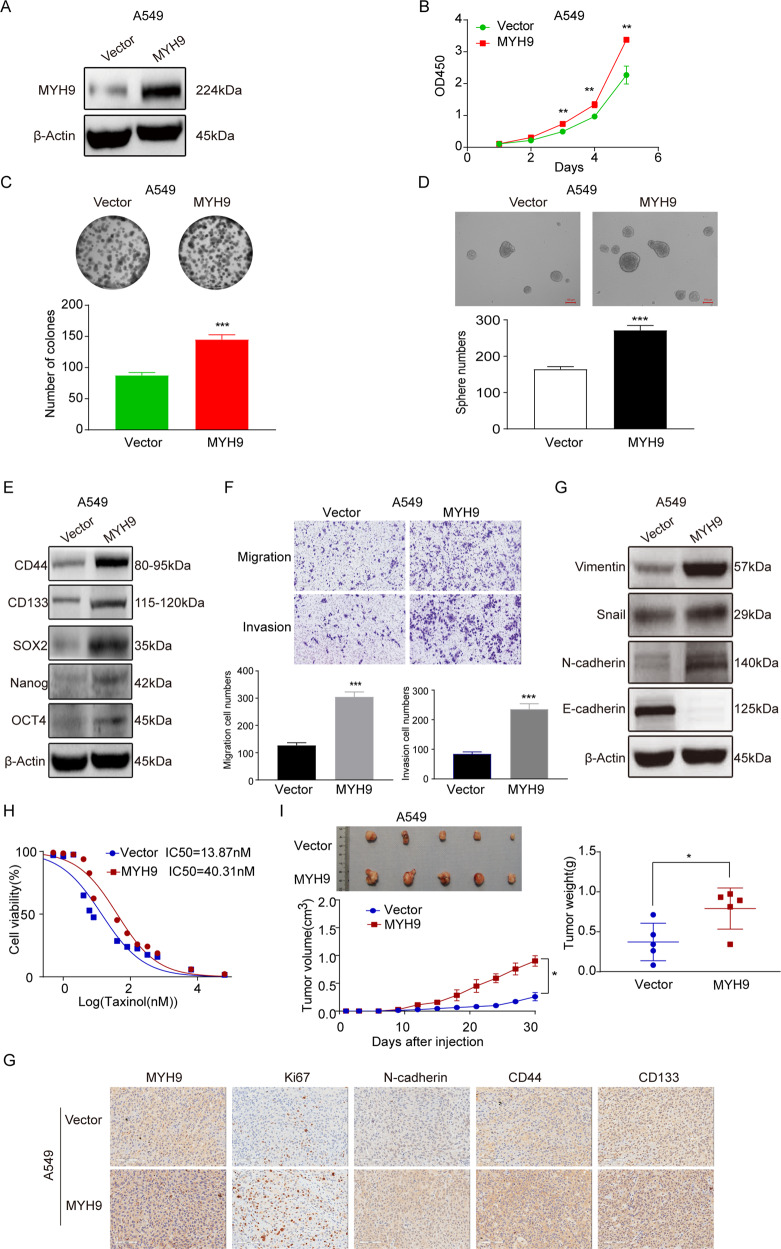
Overexpression of MYH9 enhances the stemness characteristics of LCCs in vivo and in vitro. **A** The protein expression of MYH9 in A549-MYH9 and A549-Vector cells. **B**, **C** Proliferation ability of A549-MYH9 and A549-Vector cells (mean ± SD; *n* = 3; **p < 0.01 and ***p < 0.001; Student’s *t*-test). **D** Self-renewal ability of A549-MYH9 and A549-Vector cells (mean ± SD; *n* = 3; ***p < 0.001; Student’s *t*-test). **E** The expression levels of CD44, SOX2, CD133, OCT4, and Nanog in MYH9 overexpressed and control cells. **F** Migration and invasion ability of A549-MYH9 and A549-Vector cells (scale bar, 200 μm; mean ± SD; *n* = 3; ***p < 0.001; Student’s *t*-test). **G** Expression of E-cadherin, Snail, Vimentin, and N-cadherinin A549-MYH9 and A549-Vector cells. **H** Analysis of drug resistance of MYH9-overexpressing and control cells to paclitaxel. **I** Growth of xenografts of A549–MYH9 and A549-Vector cells in nude mice (mean ± SD; *n* = 5; *p < 0.05 and **p < 0.01; Student’s *t*-test). **J** Analysis of the expression of MYH9, Ki67, N-cadherin, CD133, and CD44in transplanted tumor tissues of A549-MYH9 and A549-Vector cells (scale bar, 100 μm; *n* = 5).

A549 cell line with low endogenous expression of MYH9 has constructed a stable cell line with overexpression (Fig. 3A). Compared with the control cells, colony formation and proliferation were significantly enhanced after overexpression of MYH9 (Fig. 3B, C). Also, the self-renewal ability of MYH9 overexpression cells was significantly enhanced (Fig. 3D), and the expression levels of CD44, SOX2, Nanog, CD133, and OCT4 were significantly upregulated (Fig. 3E).

The migration and invasion ability of A549-MYH9 cells was stronger than that of A549-Vector cells (Fig. 3F), and N-cadherin, Vimentin, and Snail increased in A549-MYH9 cells (Fig. 3G). Paclitaxel resistance test showed that overexpression of MYH9 enhanced the drug resistance of LCCs (Fig. 3H). These results suggested that MYH9 can regulate the stemness characteristics of LCCs, and subsequent experiment in vivo showed that A549-MYH9 cells formed xenografts faster than A549-Vector cells, with larger volume and weight (Fig. 3I). In the xenografts with high expression of MYH9, the expression of Ki67, N-cadherin, CD44 and CD133 were upregulated (Fig. 3J).

### Blebbistatin reduced the tumorigenicity of MYH9 in vivo

**Fig. 4:**
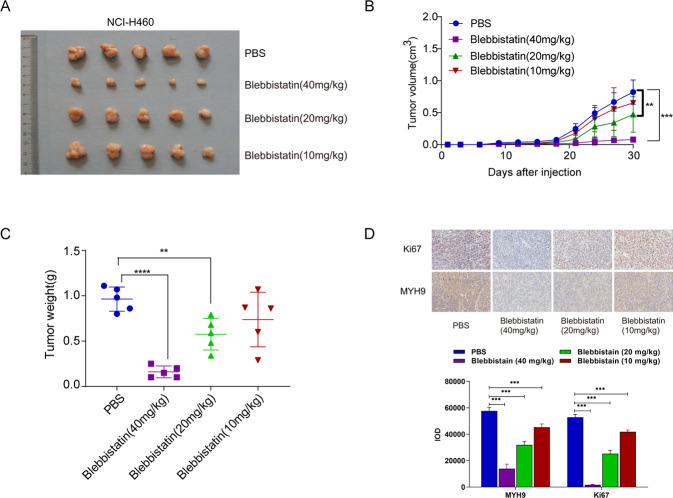
Blebbistatin reduced tumorigenicity of MYH9 in vivo. **A** Xenografts were formed in the PBS group and high, middle, and low dose (40 mg/kg, 20 mg/kg, and 10 mg/kg) groups of blebbistatin. **B** Growth curve of xenografts in different dose groups of blebbistatin and PBS group (*n* = 5; **p < 0.01 and ***p < 0.001; Student’s *t*-test). **C** Tumor weights of xenografts in high, middle, and low dose groups of blebbistatin and PBS group (*n* = 5; **p < 0.01 and ****p < 0.0001; Student’s *t*-test). **D** Expression of MYH9 and Ki67 in xenograft tissues of different dose groups of blebbistatin and PBS groups (scale bar, 100 μm; *n* = 5).

Blebbistatin is currently recognized as an inhibitor of MYH9. To further prove the effectiveness of MYH9, results revealed that the blebbistatin could significantly inhibit transplanted tumor growth and volume (Fig. 4A, B).

Simultaneously, the inhibition rates of low, middle, and high doses (10 mg/kg, 20 mg/kg, and 40 mg/kg) of blebbistatin on the tumor weight of the xenografts were 23.4%, 40.2%, and 83.4%, respectively, with a significant difference and dose-dependent effect (Fig. 4C). IHC analysis showed that the expression of MYH9 and Ki67 in the low, middle, and high dose (10 mg/kg, 20 mg/kg, and 40 mg/kg) groups of blebbistatin decreased gradually and correlated with the concentration of inhibitor (Fig. 4D). These results suggested that the blebbistatin could inhibit tumor growth in nude mice, which further proved that MYH9 can regulate the stem characteristics of LCCs.

### MYH9 regulates stem phenotype of LCCs by activating the mTOR signaling pathway

**Fig. 5:**
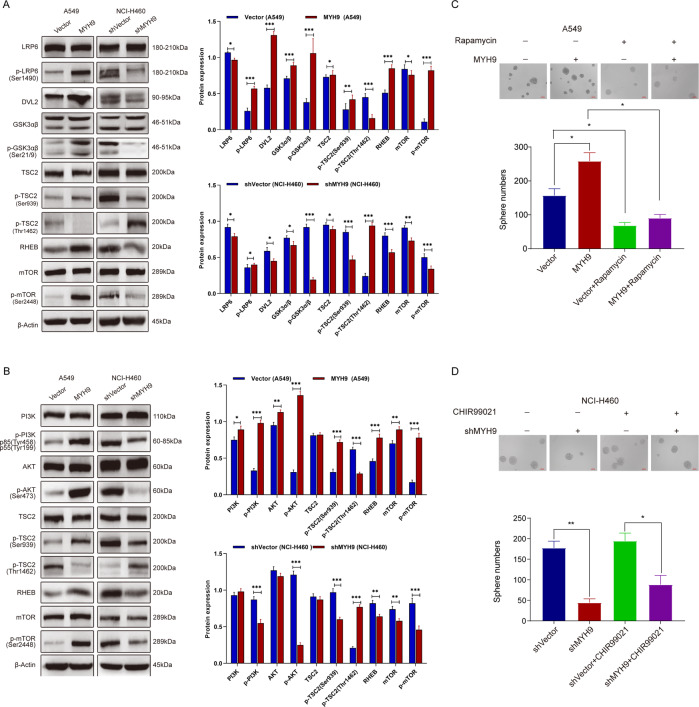
MYH9 regulates the stem phenotype of LCCs by activating the mTOR signaling pathway. **A** The protein expression of LRP6/GSK3/mTOR signal pathway markers in MYH9 stable transformant and control cells. **B** The expression of proteins from PI3K/AKT/mTOR signaling pathway in MYH9 stable transgenic and control cells. **C** Analysis of the effect of mTOR inhibitor Rapamycin on the self-renewal ability of MYH9 overexpressing cells and control cells (scale bar, 100 μm; *n* = 3; *p < 0.05; n.s., not significant; Student’s *t*-test). **D** The effect of GSK3 inhibitor CHIR–99021 on MYH9 low expression cell self-renewal ability and control cells (scale bar, 100 μm; *n* = 3; *p < 0.05and **p < 0.01; Student’s *t*-test).

To explore the specific mechanism of the effect of MYH9 on the stem phenotype of LCCs, the total proteins extracted from MYH9 knockdown and overexpression cells were used to detect the stemness-related signal pathway [32–34]. The results showed that MYH9 could activate different branch pathways of the mTOR pathway. In the LRP6/GSK3/mTOR signaling pathway, phosphorylated LRP6 in MYH9 overexpressed LCCs affected phosphorylation of GSK-3α^Ser21^ and GSK-3β^Ser9^ by upregulating DVL2. It then phosphorylated mTOR-negative regulator TSC2 and finally increased the level of mTOR phosphorylation. After MYH9 knockdown, the expression of these proteins showed the opposite trend (Fig. 5A); After MYH9 overexpression, the PI3K/AKT/mTOR signaling pathway was activated, whereas after MYH9 knockdown, the pathway was inhibited (Fig. 5B). This result indicated that MYH9 regulates the stemness of LCCs by activating the mTOR signaling pathway.

To further identify whether MYH9 acts through the mTOR signal pathway, the mTOR inhibitor Rapamycin and GSK3 inhibitor (CHIR-99021) were used to detect the self-renewal ability of LCCs. The results showed that MYH9 significantly promoted the self-renewal ability of A549 cells, while the addition of inhibitor Rapamycin blocked the effect of MYH9 on the self-renewal ability (Fig. 5C). Simultaneously, the result of MYH9 knockdown cells treated with CHIR-99021 showed that the self-renewal ability of MYH9 knockdown cells increased after the addition of inhibitor CHIR-99021 (Fig. 5D). These results suggest that MYH9 regulates the malignant stem phenotype of LCCs through the mTOR signal pathway.

### MYH9 is an independent risk factor in patients with NSCLC

**Fig. 6:**
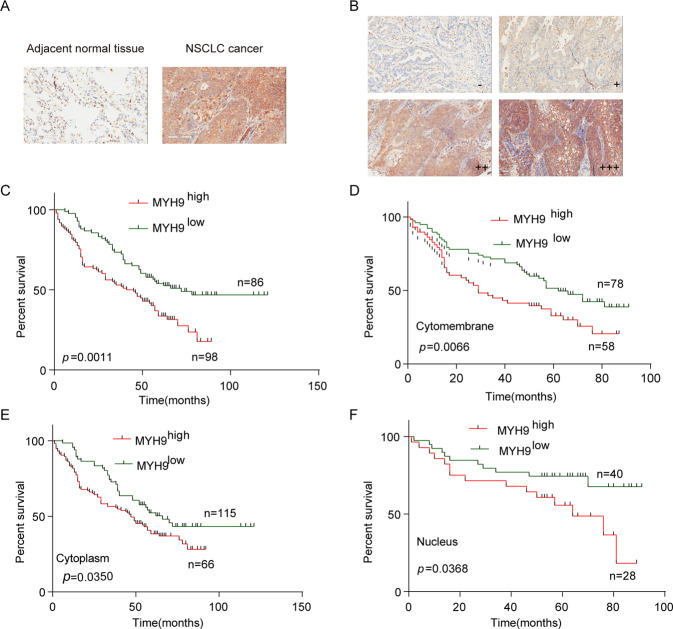
MYH9 is an independent risk factor in patients with NSCLC. **A** Expression of MYH9 in NSCLC and paracancerous tissues (scale bar, 100 μm). **B** IHC divided the expression of MYH9 in lung cancer into four grades: negative (–), weakly positive (+), positive (++), and strongly positive (+++) (scale bar, 100 μm). **C** Kaplan-Meier survival curve of overall survival in patients with NSCLC (**p < 0.01; Log-rank test). **D**, **E** Kaplan-Meier survival curve and univariate analysis were used to study the correlation between MYH9 expression in the cell membrane, cytoplasm, nucleus, and survival prognosis in patients with NSCLC (*p < 0.05and **p < 0.01; Log-rank test).

To study the clinical significance of MYH9, the expression of MYH9 in NSCLC tissue microarrays by IHC is detected. The clinicopathological features of patients with NSCLC are given in Additional file 1, schedule 1. The IHC results showed that the expression of MYH9 in NSCLC was stronger than that in matched paracancerous tissues (Fig. 6A). Also, according to the expression intensity of MYH9, patients with NSCLC were divided into four grades: negative (–), weakly positive (+), positive (++) and strongly positive (+++) (Fig. 6B). Furthermore, patients with NSCLC were divided into the MYH9^high^( + + and + ++are high) and MYH9^l^°^w^(– and + are low).The results showed that there was a significant correlation between the expression of MYH9 and the survival and prognosis of patients (*p* = 0.0011). The overall survival time of patients with up-regulated MYH9 expression (43 months) was significantly shorter than that of patients with low expression of MYH9 (72 months) (Fig. 6C), indicating that MYH9 can predict the survival time of patients with NSCLC.

MYH9 expression in the cell membrane, cytoplasm, and nucleus and survival prognosis of patients with lung cancer were 0.0066, 0.0350 and 0.0368 (Fig. 6D-F), respectively, which further confirmed that MYH9 was closely related to the prognosis of patients with NSCLC. It has the significance of predicting the survival and prognosis of patients. Simultaneously, univariate regression analysis showed that in addition to the expression of MYH9, the patient’s age, lymph node metastasis, and clinical stage were also related to the overall survival time. Multivariate Cox regression analysis showed that age, sex, clinical stage, and MYH9 expression were independent risk factors for the overall survival of patients with NSCLC, which could independently evaluate patient survival prognosis with NSCLC (Table 1).

**Table 1:** Univariate and multivariate regression analysis of prognosis in patients with NSCLC.

Features	univariate analysis		multivariable analysis	
	HR(95% CI)	*p* value	HR(95% CI)	*p* value
Age(years),≥65 v < 65	1.55(1.06–2.27)	0.023	1.73(1.16–2.56)	0.007
Gender,Male v Female	0.68(0.46–1.03)	0.068	0.57(0.36–0.91)	0.018
Tumor Volume,>18 v ≤ 18	1.04(0.70–1.53)	0.862		
LNM, Positive v Negative	2.40(1.61–3.58)	0.000		
Tumor differentiation	1.05(0.70–1.59)	0.816		
MYH9, high v low	1.91(1.30–2.8)	0.0011	2.68(1.74–4.13)	0.000
AJCC stage	2.27(1.53–3.37)	0.000	1.78(0.95–3.30)	0.009

Note: *LNM* lymph node metastasis, *HR* hazard ratio, *95% CI* = 95% confidence interval.

## Discussion

The high morbidity, high mortality, and low survival rate of patients with NSCLC are closely related to CSCs, which have the biological characteristics of unlimited self-renewal, high tumorigenicity, high invasion, and migration, and strong drug resistance [35–38]. The study found that MYH9 can regulate the stemness-related malignant characteristics of LCCs and is related to the overall survival of patients with NSCLC.MYH9, as a heavy chain of nonmuscle myosin II, is a microfilament cytoskeleton-related protein, which has various functions, such as participating in cell adhesion, migration, invasion, and regulating cell signal transduction and material transport. Through clinical tissue samples of gastric cancer, MYH9 was positively correlated with the depth of invasion and lymph node metastasis of gastric cancer, and MYH9 promoted the progression of esophageal cancer, which was closely related to lymph node metastasis and pathological grade of patients with esophageal cancer. The expression of MYH9 is upregulated in other cancers, such as colorectal cancer, head and neck cancer, and acute myeloid leukemia, and is associated with poor prognosis of these cancers [26, 27, 39–41]. Also, Arora et al. found that MYH9 can regulate the biological behavior of stem cells [30, 31]. These studies have suggested that MYH9 affects tumor progression by regulating the phenotype of CSCs.

In this study, MYH9 was enrichment expression in many kinds of LCCs from serum-free medium by flow cytometry, indicating that MYH9 can identify LCSCs and is related to the stem phenotype of tumors. Subsequently, we constructed a stable overexpression and knockdown cell line of MYH9 and studied the effect of MYH9 on stem characteristics from LCCs in vivo and in vitro. These results revealed that the ability of drug resistance, self-renewal, migration and invasion and proliferation of LCCs decreased significantly after the down-regulated expression of MYH9, and the expression levels of N-cadherin, Vimentin, E-cadherin and Snail and stem cell markers OCT4, CD133, CD44, SOX2 and Nanog also showed a downward trend. Furthermore, tumor formation tests in vivo showed that the xenografts formed by LCCs knocked down by MYH9 grew slowly, tumor size was smaller, and tumor weight was lighter, and the expression trend of metastasis molecule N-cadherin, malignant proliferation marker Ki67 and stem cell markers CD44 and CD133 in xenografts was the same as that of MYH9. In contrast, overexpression of MYH9 reversed the noted phenotypic changes. These studies suggested that MYH9 can regulate the stem phenotype of LCCs, which may be related to tumor formation and development. Subsequently, we used blebbistatin, a specific inhibitor of MYH9, to treat lung cancer in vivo, and found that after treatment with blebbistatin, the growth of LCCs was inhibited in a dose-dependent manner

To explore the molecular mechanism, we found that MYH9 plays its role by activating different branches of the mTOR signaling pathway: on the one hand, phosphorylated LRP6 affects the phosphorylation of GSK-3 by regulating DVL2 and then regulates the negative regulator of mTOR to change the level of mTOR phosphorylation; on the other hand, the expression of MYH9 affects the activation of PI3K/AKT/mTOR signaling pathway. Then, the mTOR inhibitor Rapamycin and GSK3ɑ/β inhibitor CHIR-99021 was used to verify this molecular mechanism. It demonstrated that MYH9 regulates the stem phenotype of LCCs through the mTOR signal pathway.

Finally, we found that MYH9 was highly expressed in clinical samples of NSCLC, and expression in the cell membrane and nucleus was specific to that of samples. Although some studies have shown that the expression of MYH9 is related to the histological characteristics of lung adenocarcinoma [29], more evidence is needed to prove its therapeutic value in patients with NSCLC. Univariate and multivariate analysis showed that MYH9 was positively correlated with short overall survival and poor prognosis and was an independent risk factor for patients with NSCLC.

In conclusion, our research showed that MYH9 is a potential marker of LCSC, which was enriched in spheroid cells, which affected malignant stem phenotypes of LCCs by regulating the mTOR signal pathway. The expression of MYH9 was positively correlated with the survival and prognosis of patients with lung cancer and can be used as an independent index, providing a new potential target for treating patients with lung cancer.

## Materials and Methods

### Tissue microarrays

The tissue microarrays were purchased from Shanghai Xinchao Biotechnology (China). The pathological data of clinical specimens are summarized in Tables S1. The study was approved by the medical ethics committee of Cancer Hospital, Chinese Academy of Medical Sciences (Beijing, China) (Ethical approval number: NCC1999 G-003).

### Cell culture

Cell lines A549, NCI-H1299, GLC-82, and NCI-H460 were preserved at the Laboratory of Cell and Molecular Biology, Institute of Oncology, Chinese Academy of Medical Sciences. SPCA-1 and NCI-H226 cell lines were purchased from the Shanghai Cell Bank of the Chinese Academy of Sciences. After adding fetal bovine serum (FBS) to the RPMI-1640 medium, the cells were cultured at 37 °C in 5% CO_2_. All of the cell lines were confirmed to be free of mycoplasma contamination after testing with the kit from Shanghai Yise Medical Technology (MD001).

### Self-renewal assay

The LCCs were inoculated into a low adhesion plate (Corning) with laying 500 cells in each well and cultured in a serum-free DMEM/F12 medium containing 0.8% methylcellulose added with 20 ng/ml EGF, 10 ng/mL LIF, 20 ng/mL bFGF, and B27 (1:50) factors. The fresh medium was added every three days. After 14 days, the formed spheres were photographed and counted.

### Antibodies for western blot, immunofluorescence and Immunohistochemistry

MYH9 (#M8064; Sigma), CD133 (#ab19898; Abcam), Nanog (#3580; CST), OCT4 (#2750; CST), CD44 (#3570; CST), SOX2 (#3579; CST), Vimentin (#3932; CST), Snail (#3879; CST), N-Cadherin (#13116; CST), E-Cadherin (#20874-1-AP; Proteintech), LRP6 (#2560; CST), Phospho-LRP6Ser1490(#2568; CST), DVL2 (#12037-1-AP; Proteintech), GSK-3α/b (#5676; CST), Phospho-GSK-3α/bSer21/9 (#9331; CST), Phospho-TSC2 Antibody Sampler Kit (#8350; CST), RHEB (#15924-1-AP; Proteintech), mTOR (#2983; CST), Phospho-mTORSer2448(#5536; CST), and b-Actin (#4970; CST).

### Flow cytometry

The LCCs were digested, centrifuged, and incubated with antibody MYH9 (#M8064; Sigma) at 25°C for 1 h. Then incubated with fluorescent antibody 488 (#711-486-152; Jackson ImmunoResearch;) for 0.5 h. PBS was rinsed and detected by AccuriC6cytometer (BD Biosciences).

### Proliferation, colony formation, and drug resistance assay

After the LCCs were digested, 3.5 × 10^3^ cells were cultured for 5 days, the reaction time was 2.5 h after adding 20 μL of CCK-8 assay kit (Dojindo). Finally, the absorbance at 450 nm wavelength was determined by an enzyme-labeling instrument (Bio-rad).1 × 10^3^ cells were inoculated and cultured for 14 days, then fixed, stained and dried. Finally, the clones were photographed using a microscope, and the clone formation rate was calculated. 4 × 10^3^ cells were inoculated and cultured for 24 h. Then the cells were cultured in the medium containing the concentration gradient of paclitaxe. After 72 h, add CCK-8 assay kit solution and reacted for 2.5 h. The OD450 was detected by an enzyme-labeling instrument.

### Migration and invasion analysis

The medium without FBS and Matrigel (BD Biosciences) were added to the Transwell chamber (8-μm pore size, Millipore). The 3×10^4^ cells were inoculated with serum-free medium in the upper chamber of the plate. Finally, five visual fields for each well were counted under the microscope. In the migration assay, Matrigel was not added in the chamber, the other steps were the same as in the invasion assay.

### Tumorigenicity and treatment in nude mice

Female BALB/c nude mice (4-5 weeks old) were purchased from Huafukang Company. For tumorigenesis test, 3×10^6^ cells were injected into each nude mice. The animals were killed when the tumor’s length and diameter were 1–2 cm, and the tumor was photographed and weighed.

For blebbistatin treatment test, 3 × 10^6^ cells were inoculated under the axilla of the forelimbs of mice. Nude mice were divided into four groups: PBS group, high-dose group (40 mg/kg), medium-dose group (20 mg/kg), and low-dose group (10 mg/kg); 0.4 mL blebbistatin was injected intraperitoneally in each nude mice. The nude mice were treated once every other day for four weeks. Other steps were the same as tumorigenesis assay.

### Statistical analysis

Analysis of variance (ANOVA) or Student’s *t*-test was used to compare the results between experimental data groups, and the value was expressed as means ± SD. Spearman analysis compared the correlation between the two; Fisher exact test and chi-square test compared the correlation between clinical parameters; Cox proportional hazard regression model Log-rank test and Kaplan-Meier were used for survival analysis statistical. GraphPad Prism7.0 and SPSS software were used for data analysis, and the statistically significant standard value was *p* < 0.05.

## Supplementary information

Table S1

The Ethics approval

## Data Availability

The datasets generated during and/or analysed during the current study are available from the corresponding author on reasonable request.
