# Supplementary material for: MYH9 is crucial for stem cell-like properties in non-small cell lung cancer by activating mTOR signaling
Source: Cell Death Discov. 2021 Oct 11;7:282. doi: 10.1038/s41420-021-00681-z (PMC8505404; doi:10.1038/s41420-021-00681-z)
Supplement: Supplementary file 1 — Table S1 [file 41420_2021_681_MOESM1_ESM.docx]

Tables S1 Clinicopathological data of tissue specimens of NSCLC

| **Characteristics** | **No. of patients** | **%** |
| --- | --- | --- |
| **Age(years)** |  |  |
| <65 | 103 | 56 |
| ≥65 | 79 | 42.9 |
| Missing data | 2 | 0.01 |
| **Gender** |  |  |
| Female | 49 | 26.6 |
| Male | 135 | 73.4 |
| **Tumor Volume(cm^3^)** |  |  |
| ≤18 | 73 | 39.7 |
| >18 | 106 | 57.6 |
| Missing data | 5 | 2.7 |
| **AJCC stage** |  |  |
| Stage Ⅰ | 55 | 29.9 |
| Stage Ⅱ | 52 | 28.3 |
| Stage Ⅲ | 64 | 34.8 |
| Stage Ⅳ | 3 | 0.02 |
| Missing data | 10 | 0.05 |
| **Lymph node status** |  |  |
| Negative | 92 | 50 |
| Positive | 86 | 46.7 |
| Missing data | 6 | 0.03 |
| **Tumor differentiation** |  |  |
| Well | 20 | 10.9 |
| Moderate | 109 | 59.2 |
| Poor | 55 | 29.9 |
| **Pathological type** |  |  |
| LSC | 90 | 48.9 |
| LAC | 94 | 51.1 |
