# Supplementary material for: MYH9 is crucial for stem cell-like properties in non-small cell lung cancer by activating mTOR signaling
Source: Cell Death Discov. 2021 Oct 11;7:282. doi: 10.1038/s41420-021-00681-z (PMC8505404; doi:10.1038/s41420-021-00681-z)
Supplement: Supplementary file 2 — The Ethics approval [file 41420_2021_681_MOESM2_ESM.pdf]

中国医学科学院肿瘤医院
伦理委员会

国家973 重点基础研究项目申报
伦理审查批文

致有关部门：

中国医学科学院肿瘤医院伦理委员会于1999年2月10日下午召开伦理审查会议（会议地址：病房楼二层会议室），对我院杨治华同志申报的“胃癌侵袭转移功能基因的生物学特征及分子机制研究”进行了审批（审批编号：【NCC1999 G-003】）。伦理委员通过认真审查认为该项目基本符合伦理要求，相关文件符合伦理原则，同意开展相关临床样本的研究。

（具体投票结果可向伦理委员会查询，联系人：赵秀娟，电话：87788495）

中国医学科学院肿瘤医院

伦理委员会

1999年2月10日
